# Flavonoid engineering of flax potentiate its biotechnological application

**DOI:** 10.1186/1472-6750-11-10

**Published:** 2011-01-28

**Authors:** Magdalena Żuk, Anna Kulma, Lucyna Dymińska, Katarzyna Szołtysek, Anna Prescha, Jerzy Hanuza, Jan Szopa

**Affiliations:** 1 Faculty of Biotechnology, University of Wrocław, Poland; 2 Institute of Chemistry and Food Technology, Faculty of Economics and Engineering, University of Economics, Wrocław, Poland; 3 Department of Food Science and Nutrition, Wroclaw Medical University, Nankiera 1, 50-140 Wrocław, Poland; 4 Institute of Low Temperatures and Structure Research, Polish Academy of Sciences, Wrocław, Poland; 5 Linum Fundation, Stablowicka 147/149,54-066 Wroclaw, Poland

## Abstract

**Background:**

Flavonoids are a group of secondary plant metabolites important for plant growth and development. They show also a protective effect against colon and breast cancer, diabetes, hypercholesterolemic atherosclerosis, lupus nephritis, and immune and inflammatory reactions. Thus, overproduction of these compounds in flax by genetic engineering method might potentiate biotechnological application of these plant products.

**Results:**

Flax plants of third generation overexpressing key genes of flavonoid pathway cultivated in field were used as plant material throughout this study. The biochemical properties of seed, oil and seedcake extracts and fibre from natural and transgenic flax plants were compared. The data obtained suggests that the introduced genes were stably inherited and expressed through plant generations.

Overproduction of flavonoid compounds resulted in increase of fatty acids accumulation in oil from transgenic seeds due to protection from oxidation offered during synthesis and seed maturation. The biochemical analysis of seedcake extracts from seeds of transgenic flax revealed significant increase in flavonoids (kaempferol), phenolic acids (coumaric, ferulic, synapic acids) and lignan content. Fibres, another product of flax plant showed increase in the level of catechine and acetylvanillone and decrease in phenolic acids upon flax modification.

Biochemical analysis results were confirmed using IR spectroscopy. The integral intensities of IR bands have been used for identification of the component of phenylpropanoid pathway in oil, seedcake extract and fibre from control and transgenic flax. It was shown that levels of flavonoids, phenolic acids and lignans in oil and seedcake extract was higher in transgenic flax products compared to control. An FT-IR study of fibres confirmed the biochemical data and revealed that the arrangement of the cellulose polymer in the transgenic fibres differs from the control; in particular a significant decrease in the number of hydrogen bonds was detected.

**Conclusions:**

All analysed products from generated transgenic plants were enriched with antioxidant compounds derived from phenylopropanoid pathway Thus the products provide valuable source of flavonoids, phenolic acids and lignan for biomedical application. The compounds composition and quantity from transgenic plants was confirmed by IR spectroscopy. Thus the infrared spectroscopy appeared to be suitable method for characterization of flax products.

## Background

Flax (*Linum usitatissimum*) plant has a long history of traditional use both as a source of oil and fibre and is grown for commercial use in over 30 countries of the world. In Poland, flax is the most important industrial oil and fibre crop.

Flax seeds have long been used in human and animal diet and in industry as a source of oil and as the basal component or additive of various paints or polymers. Recently there has been a growing interest in the probiotic properties of flax and in its beneficial effects on coronary heart disease, some kinds of cancer and neurological and hormonal disorders [1-3]. The beneficial effects are mostly due to flax lipids. Flax oil is the richest plant source of linoleic and linolenic polyunsaturated fatty acids (PUFA), which are essential for humans since they cannot be synthesized in the organism and must be ingested with food.

Unfortunately, essential fatty acids are highly susceptible to oxidation and flax oil therefore has a very short shelf life. Only certain cultivars (e.g. Linola) with appropriate lipid composition are suitable for commercial preparation of edible oil [4,5]. In flax grains, lipids are suitably protected against oxidation by various mechanisms and the antioxidative effect of phenylpropanoids which are present in seedcake is among them. However, even after cold extraction most of these mechanisms are no longer operative and phenylpropanoids as hydrophilic compounds are not effectively extracted with oil, remaining associated with seedcakes.

Therefore seedcake, which is defatted seed, might appear as a good source of easily extracted compounds of phenylpropanoids pathway with antioxidant activity. The antioxidant compounds extracted from seedcake might have potential application in medicine. It was already suggested the beneficial role of kaemferol and quercetin as well as lignans for human in preventing against different types of cancer, cardiovascular diseases and diabetes. Very recently the extract from seedcakes was successfully used as a component of new bandage for healing of human chronic ulceration [6,7].

To avoid a fast appearance of rancidity, flax oil is often cold-pressed, supplemented with vitamin A and E or stored in dark glass jars. Since none of these protection methods are fully satisfactory, further improvements are looked for. Genetic engineering approach could involve the overproduction of various natural antioxidants within flax grains. In addition to preventing fat rancidity, antioxidants such as flavonoids might also have beneficial effect on human health.

Plant phenylpropanoids are very broad group of biochemical compounds, which form secondary metabolites in the enzymatic biosynthesis. They include flavonoids, phenolic acids, phenols, lignans and tannins [8-10]. Flavonoids are involved in many biochemical processes of plant growth and development. They act as antioxidants, chelators of divalent cations [11] photoreceptors and visual attractors [12] They protect plants against pathogenic micro-organisms [13], herbivores, UV radiation [14] and oxidative and heat stresses. Their antioxidant activity influences the food quality due to their inhibitory action on enzymatic and non-enzymatic peroxidation [15]. Flavonoids also exhibit anti-allergic, antiviral, anti-inflammatory and vasodilatory activities [12,16].

Thus the aim of our previous study was to increase antioxidant potential (via overexpression of regulatory genes of phenylpropanoid pathway) of flax for greater accumulation of PUFA and its higher stability against oxidation. Indeed simultaneous overexpression of chalcone synthase (CHS), chalcone isomerase (CHI) and dihydroflavonol reductase (DFR) resulted in significant accumulation of flavonoids in seeds extract and thus the increase stability of unsaturated fatty acids against oxidation was detected [17,18].

The aim of this work was to analyze separately oil and seedcake extracts as well as fibers for the presence and content of biologically active compounds by liquid chromatography and IR spectroscopy in order to determine their possible applications. Simultaneous use of those two techniques allow for a obtaining of much more complete data. When liquid chromatography (UPLC) is satisfactory for identification and measurements of quantity of compounds, however it does not always provide data on the structure of newly synthesized phenylpropanoids, their potential modification and their presence in fiber and oil. The last was of special interest for the reason that hydrophilic flavonoids are present in oil in trace amount and therefore their content and structural analysis in respect to their antioxidative role might be biotechnologically useful. Therefore FT-IR spectroscopy was used in the characterization of seed oil, seedcake extracts and fibres for their chemical composition and molecular changes.

## Methods

### Transgenic flax generation and selection

#### Plant material

Flax seeds (cv. *Linola*) were obtained from The Flax and Hemp Collection of the Institute of Natural Fibres, Poland. Initially the transgenic and control plants were grown in tissue culture and were cultivated in a greenhouse under a 16 h light (21°C), 8 h darkness (16°C) regime. Next the plants were grown in soil in individual pots and were watered daily. Finally the seeds were used for plant propagation in a field. In this work third generation of transgenic seeds were grown in a field, and seeds were harvested 4 months after the transfer of the seeds to the soil.

#### Transgenic plant construction and selection

Two-week old cotyledon and hypocotyl explants were transformed. For transformation, we used the binary vector containing three cDNAs from *Petunia hybrida*, encoding chalcone synthase (CHS, EMBL/GenBank database acc. no. X04080), chalcone isomerase (CHI, EMBL/GenBank database acc. no. X14589) and dihydroflavonol reductase (DFR, EMBL/GenBank database acc. no. X15537) in the sense orientation under the control of the 35S promoter and OCS terminator [19].

The vector was introduced into *Agrobacterium tumefaciens *strain C58C1: pGV2260. *A. tumefaciens*-inoculated explants were subsequently transferred to a callus induction and shoot regeneration medium [20].

The transgenic plants were preselected *via *PCR using primers specific for the kanamycin resistance gene (*npt II*), and then selected by means of northern blot analysis. PCR was carried out using specific primers for the neomycine phosphotransferase gene (forward, CCGACCTGTCCGGTGCCC; reverse, CGCCACACCCAGCCGGCC) on genomic DNA isolated from 3-week old tissue-cultured plants as a template.

Total RNA was prepared from frozen young leaves using the guanidinium hydrochloride method [21]. Total RNA was separated on an agarose gel (1.5% (w/v) agarose, 15% (v/v) formaldehyde) and blotted onto a Hybond N + (Amersham) filter. The membrane was hybridized overnight at 42°C with radiolabeled cDNAs (CHS, CHI, and DFR) as probes and then washed three times with SSPE buffer containing 0.1% SDS for 30 min at 42°C.

The details on plant transformation, selection and transgenic plant analysis was described previously [17].

### Oil and seedcake preparation

25 kg of flax seeds was used for pressing of oil on industrial worm gear oil press (Oil PressDD85G - IBG Monoforts Oekotec GmbH& Co). Flax seeds were grounded and transferred to press machine for cold pressing of oil. The mean yield of procedure is 25% oil and 75% seedcakes. Oil and seedcakes were collected and used for further experiments.

### Biochemical analysis of oil

#### Determination of fatty acids content in oil

Methyl esters of fatty acids (FAMEs) were extracted from oil using 0.5M KOH in methanol. After that sample was neutralized using 1.25M HCl in methanol. Then methyl esters of fatty acids were extracted into hexane. The hexane phase was collected; the lipids were concentrated in N_2 _stream and stored at -20°C. The methyl esters were quantified by gas chromatography (Agilent Technology 6890N with FID detector), using pentadecanoic acid as an internal standard [22].

#### Determination of phenolic compounds in oil

Total phenolic compounds was measured using Folin-Ciocalteu method [23] in methanol extracts (90% v/v) from oil. The phenolic compounds content was calculated as equivalents of caffeic acid.

#### Determination of tocopherols, plastochromanol-8 and β-carotene

Tocopherols and plastochromanol-8 and β-carotene contents were determined by high-performance liquid chromatography (HPLC- Waters Milford 600 with fluorimetric detector, excitation 290 nm, emission 330 nm) with β-tocopherol as internal standard. The samples were first analyzed without internal standard to confirm the absence of β-tocopherol. For analysis of β-carotene the UV-VIS detector (450 nm) was used [24].

### Antioxidant potential of oil

#### Peroxide value measurement in oil

The peroxide value is determined by measuring the amount of iodine which is formed by the reaction of peroxides (formed in oil) with iodine ion. Peroxide value was measured as content of mol/dm^3 ^sodium thiosulfate (Na_2_S_2_O_3_).

#### TBARS measurements in oil

The level of TBARS (thiobarbituric acid-reactive substances) was measured according to the published protocol [18]. Oil samples (4 μl) were oxidized at 140°C for 40 min in tightly closed glass test tubes, using laboratory oven. After the initial baking time, 2 ml of reagent (15% TCA and 0.37% TBA in 0.25 M HCL) was added to each sample, and the mixture was thoroughly blended. Then the test tubes were heated at 100°C for 15 min and cooled under running tap water. After a 10-min centrifuge, the absorbance at 535 nm was measured.

### Preparation and biochemical analysis of seedcake extracts

#### HPLC analysis of flavonoid glycoside content

The materials used in this study were crushed using a laboratory mill. A 1 g sample of flax seedcakes was extracted with 7 ml 35% aqueous methanol containing 1 g/L L-ascorbic acid as an antioxidant, for 18 h at 20°C in glass screw-capped vials, and then sonicated for 15 min. Next, the samples were centrifuged (5 min, 19,000 g) and the clear supernatant was injected onto a HPLC column. The analysis of flavones and flavonols derivatives were carried out on a Merck-Hitachi L-7455 liquid chromatograph with a diode array detector (DAD) and quaternary pump L-7100 equipped with D-7000 HSM Multisolvent Delivery System (Merck-Hitachi, Tokyo, Japan) and an L-7200 auto sampler. Separation was performed on a Synergi Fusion RP-80A 150 × 4.6 mm (4 μm) Phenomenex (Torrance, CA, USA) column. The oven temperature was set to 20°C. The mobile phase was composed of solvent A (2.5% acetic acid) and solvent B (acetonitrile). The program began with a linear gradient from 0% B to 25% B at 36 min, followed by washing and reconditioning of the column. The flow rate was 1.0 mL/min, and the runs were monitored at the following wavelengths: flavones at 340 nm and flavonols derivatives at 360 nm.

#### Determination of the total anthocyanin glycoside content *via *the pH-differential method

15 mg of seedcakes was extracted with 1 mL of methanol/HCl (95:5, v/v) in an ultrasonic bath for 30 min. The extract was centrifuged at 14,000 g for 10 min. Two dilutions of the sample were performed: first, 100 μL of the supernatant was mixed with 900 μL of 0.025 M potassium chloride buffer, pH 1.0, and then, 100 μL of supernatant was mixed with 900 μL of 0.4 M sodium acetate buffer, pH 4.5. The solution was allowed to stand at RT for 15 min, and then the absorbance at 510 nm and 700 nm was measured, which allowed for haze correction. The results were reported as cyanidin-3-*O*-glucoside equivalents

#### Preparation of alkali hydrolyzed seedcake extract

Flax seedcakes (100 g) were extracted three times with 400 ml of 80% methanol (v/v) for 15 min at 80°C. The extract was centrifuged, pellet discarded and methanol from supernatant fraction evaporated at 40°C. The aqueous fraction of extract was subjected to alkaline hydrolysis in final concentration 0.3 M sodium hydroxide for 2 days at room temperature followed by neutralization using 2M hydrochloric acid. Neutralized extract was centrifuged and supernatant was sterilized by filtration through Acrodisc (Gelman Sciences, Ann Arbor, MI) 0.22 μm filter or by autoclaving at 120°C for 20 min [25].

#### Secoisolariciresinol diglucoside (SDG) content measurement

The seedcake extract after alkali hydrolysis was analyzed on Waters Acquity UPLC System with a 2996 PDA detector, using Acquity UPLC column BEH C18, 2.1 × 100 mm, 1.7 μm. The mobile phase of A = acetonitrile/B = 20 mM ammonium formate pH 3 in a gradient flow: 1 min.10%/90% A/B, 2-10 min. gradient to 30%/70% A/B, 12 min gradient from 30% to100% A, and 13 min gradient from 100%A to 10%/90% A/B with a 0.4 mL/min flow rate. The compound content was measured at 320 nm [25].

#### Phenolic acid content measurement

The seedcake extract after alkali hydrolysis was analyzed on Waters Acquity UPLC System with a 2996 PDA detector, using Acquity UPLC column BEH C18, 2.1 × 100 mm, 1.7 μm. The mobile phase of A = acetonitrile/B = 20 mM ammonium formate pH 3 in a gradient flow: 1 min. 10%/90% A/B, 2-6 min. gradient to 40%/60% A/B, 7 min gradient from 40% to100% A, and 8 min gradient from 100%A to 10%/90% A/B with a 0.4 mL/min flow rate. The compound was measured at 280 nm [25].

### Antioxidant capacity of seedcake extract

The chemiluminescence method was used to determine the antioxidant activity of the extracts. A methanol (100%) extract of flax was diluted in the range of 1000-15.000 times with water, and directly analyzed according to the published method of Lukaszewicz et al. [26]. This experiment was performed in a final volume of 250 μL on white microplates in a solution containing 0.1M Tris-HCl buffer, pH 9.0, and 4 mM 2.2-azobis (2-amidinopropane) dihydrochloride (AAPH), freshly prepared. The luminol solution (100 mM) and diluted extract were automatically injected. The photons produced in the reaction were counted on an EG&G Berthold LB96P microplate luminometer at 30°C. The antioxidant potential (IC50) was defined as the amount of flax extract (mg DW) that inhibits luminol chemiluminescence by 50%.

### Preparation and biochemical analysis of flax fibers

#### **Flax retting**

Control and transgenic plants were grown in a field in the vicinity of Wroclaw and harvested after 4 months. Those plants were then retted by the dew method as described [27]. Briefly, plants were spread out in a field for at least 40 days with the plants being turned every 2 weeks. During this process, bacteria and fungi grew on the plants and caused degrading of the cell-wall polysaccharide and middle lamella. Due to this process fibers were released from the stems.

#### Extraction of phenylopropanoids from fibers

1 g of flax fibers were grounded in a Retch mill to a fine powder and extracted trice with methanol. Extracts were pooled, evaporated under vacuum and resuspended in 2 ml methanol.

The remaining matter was hydrolyzed in 2 N NaOH at room temperature for 24 h in order to release bound phenolics. Extracts were adjusted to pH 3, dried under vacuum and resuspended in 2 ml of methanol.

#### UPLC analysis of phenolics

The components were analyzed using the Acquity UPLC system (Waters) equipped with an automated sample injector and PDA detector. A 10 μl sample was applied to an Acquity UPLC HSS- T3 column (2.1 × 100 mm, 1.8 μm) retaining better hydrophilic components. The mobile phase was passed through the column at a flow rate of 0.5 ml/min. The mobile phase consisted of the following components. A: 0.1% formic acid; and B: 100% methanol. For the first 2 minutes, isocratic elution was carried out using 100% of A. From 2 to 5 minutes, a linear gradient was applied using 100 to 30% A in B. From 5 to 5.5 minutes, a linear gradient was applied using 30 to 0% A in B. In the final minute concentration of A was returned to 100%.

The additional analysis of very hydrophilic component was performed using UPLC HILIC (2.1 × 100 mm, 1.7 μm) column. The mobile phase was passed through the column at a flow rate of 0.4 ml/min. The mobile phase consisted of the following components. A: 0.1% formic acid; and B: 100% acetonitrile. For the first 4 minutes, isocratic elution was carried out using 10% of A in B. From 4 to 8 minutes, a linear gradient was applied using 10 to 90% A in B. From 8 to 9 minutes, a linear gradient was applied using 90 to 100% A in B. In the final minute concentration of eluting solvents was returned to 10% of A in B.

#### Cellulose content analysis

Using the colorimetric method with anthron reagent, the cellulose content was determined for fibers obtained from the field-cultivated control and transgenic plants. For the sequential release of lignin, hemicelluloses and xylosans, stem samples were incubated in a mixture of nitric and acetic acid (1:8, v/v, for 30 min at 100°C) and then centrifuged. The resulting pellet was washed twice with water (0.5 ml) and resuspended in 1 ml of 67% H2SO4 (v/v). After tenfold dilution with fresh anthrone reagent, and incubation at 100°C for 15 min, the cellulose level was determined spectrophotometrically at 620 nm against cold anthrone reagent [28].

#### Lignin content measurement

The total lignin content in the fibers was determined via the acetyl bromide method. Lignins were isolated from fibers obtained from the field-grown control and transgenic plants. 10 ml water was added to dried fibers (10-15 mg), and these samples were heated for 1 h at 65°C and stirred every 10 min. Then, the samples were filtered through a GF/A glass fiber filter and rinsed with each of the following solutions: water, ethanol, acetone and diethyl ether. The filters were placed in glass vials and heated overnight at 70°C. After that, 25% acetyl bromide

(2.5 ml) was added to each vial, and the vials were kept at 50°C for 2 h. The cooled samples were mixed with 10 ml of 2 N sodium hydroxide and 12 ml of acetic acid. The samples were left overnight, and the lignin content was analyzed by the UV method and measured at 280 nm. Coniferyl alcohol was used to prepare a calibration curve, and the results are reported as equivalents of coniferyl alcohol [29]

#### Determination of the pectin content

Pectins were measured using modified method described by Melton and Smith [30]. Prior to pectin extraction the fibers were extracted with different solvent to remove the lipids and soluble sugars. The samples were washed with 96% ethanol at 100°C, centrifuged (5000×g, 5 min), and the supernatant was removed. The pellet was washed with 80% ethanol at 80°C, and subsequently treated with mixture of chloroform and methanol (1:1 v/v). After successive centrifugation (5000 g, 5 min) the pellet was washed with acetone and centrifuged once more (1000.g, 5 min). The supernatant was discarded and the remaining pellet dried at 37°C, frozen, and weighed. Measurement of total pectin was performed using colorimetric method after acidic hydrolysis. For hydrolysis, 100 μl of concentrated sulphuric acid was added to each sample (no more than 5 mg of dried, frozen pellet remaining after previous steps). The samples were stirred for 5 min at 4°C. The volume of each sample was next adjusted to 1 ml with distilled water in three steps (50 μl, 250 μl. and 700 μl of water). The samples were stirred after each addition of water for 5 min at 4°C. The pectin content was measured 7 spectrophotometrically at 520 nm using biphenyl method. Galacturonic acid was used for the calibration curve.

#### IR spectra

The IR spectra at room temperature were measured in the spectral range 50-4000 cm^-1 ^using a FT-IR Biorad 575C spectrometer with a 2 cm^-1 ^resolution. The control and transgenic flax products were powdered in a automatic mortar grinder. In the mid-IR region, the samples were prepared in KBr pellets, and in the far-IR region, in Nujol suspension.

The mathematical deconvolution of the spectral contours into Lorenz components was made using Origin Scientific Graphing and Analysis Software in the 7.5 version.

## Results

### Transgenic Plant Generation and Selection

The hypocotyl and cotyledon explants of flax plants were transformed with a multigene vector containing three cDNAs encoding key enzymes of flavonoid biosynthesis, according to the *Agrobacterium *method [20]. The construct consisting of *CHS*, *CHI *and *DFR *cDNAs from *Petunia hybrida *under the control of CaMV 35S promoter and OCS terminator, was inserted into the genome of the flax plants [19]. The obtained regenerants were prescreened using the PCR method with specific primers for the neomycin phosphotransferase gene. Plants that exhibited a 475-bp (*npt II*) DNA fragment were used for further selection by means of northern blot analysis. Densitometry of northern blot revealed the significant increase of mRNA for all introduced genes compared to control and specifically for CHS 4.9 and 5.3-fold for W92.40 and W92.72 respectively; for CHI 5.9 and 5.4-fold for W92.40 and W92.72 respectively; and for DFR 1.16 and 1.7-fold for W92.40 and W92.72 respectively. Details on plant selection were published previously [17].The two transgenic lines (W92.40 and W92.72) that showed the highest level of mRNA for the three introduced cDNAs were used for further analysis.

Throughout this study plants of third generation (homozygous lines) cultivated in field in a 2008 season were used. Based on PCR analysis of total RNA there were no differences in expression of three introduced genes when compared to plant of first generation (not shown). There were also no visible differences in transgenic plant phenotype when comparing to control. The leaf shape and size or petal and seed color were the same as for non-transformed plants. However, the obtained transgenic plants showed a higher productivity of seeds compared to the control plants. Transgenic plants produced 60% more seeds per ha than non-transformed plants.

### Biochemical analysis of seed

For the complete analysis of seeds from field grown plant flavonoids content was determined. Our earlier experiments have shown significant changes in the level of flavanones, flavonols (kaempferol, quercetin) and anthocyanin in transgenic flax upon overexpression of CHS, CHI and DFR cDNAs. Therefore we analyze those compounds level in extract from seeds of F3 plant generation to verify the stability of their production.

**Figure 1:**
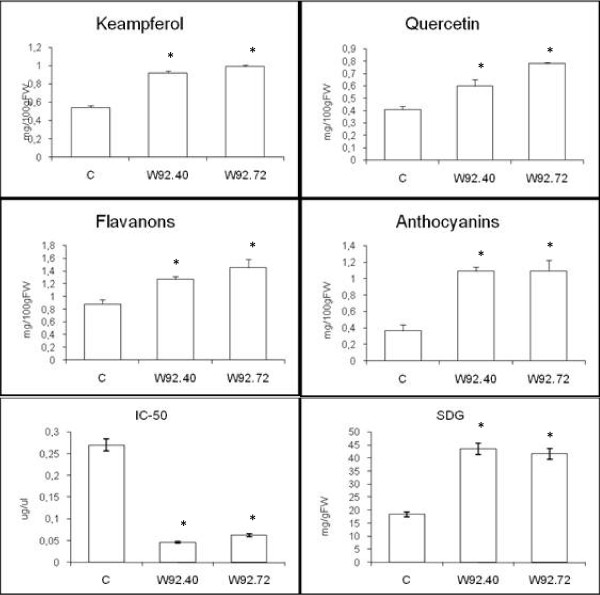
**Biochemical analysis of seeds**. The content of kaempferol and quercetin glycoside. total anthocyanins, flavanons and SDG content in seeds from control (C) and the transgenic flax plants (numbered). The antioxidant activity of plant extracts is presented as IC-50 parameter. The mean value (n = 6) ± SD for seeds from plant cultivated in a field is presented. *Statistically significant (P < 0.05)

HPLC analysis of seeds methanol extract from W92.40 and W92.72 transgenic lines reveals increase in the production of quercetin derivative (46% and 90%, respectively); moreover, analysis reveals increase in kaempferol derivative accumulation in seeds by about 70% in line W92.40 and 83% in line W92.72( Figure 1).

Since the third gene in our transgenic construction was *DFR*, the product of its activity (anthocyanins) was also measured. We found an increase in antocyanins level of about 198% in the seeds (for line W92.40 and W92.72 -197% and 199%, respectively).(Figure 1).

Flavonoids were shown to exhibit antioxidant properties [11]. Very important for health promoting properties of flax seeds are lignans which are known as effective anti-cancer factor. The level of a main flax lignan, secoisolariciresinol diglucoside (SDG)was more than two times higher in lines W92.40 and W92.72. Since there was an increase in the total phenolic compounds detected in transgenic plants, an increase in antioxidant capacity was expected. The antioxidant properties of flax seeds extract were assessed *via *the chemiluminescence method, as described previously [31]. The antioxidant potential is expressed as the IC_50 _value, which means the amount of seed extract inhibiting the oxidation of luminol by 50%. The antioxidant properties of the seeds of transgenic plants were higher than those of the non- transformed plant. The 6-fold increase of antioxidant capacity in line W92.40 and 4-fold in line W92.72 was observed. Figure 1 shows the antioxidant level of the seed extract from transgenic and control plants. This data indicates that the antioxidative status of the transgenic plants mainly resulted from the activation of the flavonoid biosynthesis pathway, through the overexpression of the three key flavonoid genes.

The overall conclusion that can be drawn from biochemical analysis of transgenic seeds is that after three seasons (2006-2008) of plant cultivation in field the level of producing compounds as the result of three genes overexpression is stable. Thus it is suggested that introduced *Petunia *genes into the flax are stably inherited and expressed.

### Preparation of oil from seeds

The seeds from plant field grown in the 2008 season were used as a source of oil. The oil was produced by typical industrial method including seeds grounding and cold pressing. There was no difference in the yield of oil from control and transgenic seeds and in both cases was about 25%.

### Biochemical analysis of oil from seeds

The objective of this work was to analyze fatty acids content and composition, tocopherols, plastochromanol-8 and β-carotene as the known flax compounds with chemotaxonomic significance [24]. For fatty acids composition and quantity the flaxseed oil was examined by gas chromatography equipped with FID (flame ionization) detector. Tocopherols, plastochromanol-8 and β-carotene were assayed by HPLC analysis.

It was expected that overproduction of flavonoids in plant might improve the fatty acids composition in seeds. Indeed the data presented in Table 1 shows the increase in the content of fatty acids in oil. The increase in unsaturated 18:1 (6%), 18:2 (38-49%) and 18:3 (48-61%) fatty acids in both transgenic lines was detected. The highest increase was measured in W92.40 transgenic line.

**Table 1:** Fatty acids composition of oil from Linola (control) and transgenic seeds (W92.40 and W92.72) expressed in μg/gFW.

	16:0	16:1	16:2	16:3	18:0	18:1	18:2	18:3	20:0	20:1	22:0	22:1	24:0	Total
Linola	11.33 ± 1.8	0.16 ± 0.05	0.12 ± 0.04	0.10 ± 0.0	6.38* ± 0.8	32.57 ± 1.8	158.57* ± 11.3	3.64 ± 1.8	0.26 ± 0.1	0.19 ± 0.4	0.12 ± 0.3	0.10 ± 0.0	0.03 ± 0.0	213.57 ± 22.8

W92.40	14.68* ± 1.5	0.27 ± 0.02	0.18 ± 0.01	0.15 ± 0.0	7.32* ± 0.5	34.55* ± 1.3	236.55* ± 9.4	5.86* ± 1.1	0.16 ± 0.1	0.30* ± 0.2	0.15 ± 0.1	0.20 ± 0.1	0.05 ± 0	300.42* ± 18.6

W92.72	13.75* ± 0.9	0.26 ± 0.09	0.18 ± 0.02	0.12 ± 0.0	6.49 ± 0.4	34.65* ± 1.5	218.51* ± 15.1	5.39* ± 1.4	0.18 ± 0.1	0.29* ± 0.5	0.13 ± 0.5	0.15 ± 0.0	0.05 ± 0	280.14* ± 21.8

The mean value (n = 6) ± SD for oil from plant cultivated in a field is presented.*Statistically significant (P < 0.05)

Beside composition the higher accumulation of all fatty acids in transgenic seeds when compared to the control was observed. The 31% and 40% increase of total fatty acids content in oil from transgenic plant W92.40 and W92.72, respectively, compared to the control. Thus it is suggested that accumulation of flavonoids in flax reasonably affect the fatty acids production in seeds.

**Figure 2:**
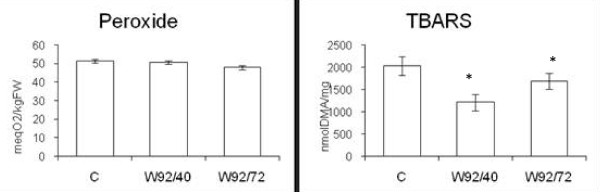
**Antioxidant potential of oil**. The peroxide value, TBARS in heated oils from control(C) and transgenic plants (numbered). The mean value (n = 6) ± SD for oil from plant cultivated in a field is presented. *Statistically significant (P < 0.05)

The next step in oil analysis was assessment of its stability. Level of lipid oxidation stability was determined by by measuring peroxide value (level of prime products of lipid peroxidation) and TBARS (indication of malonic dialdehyde - MDA the one of secondary products of lipid peroxidation) ( Figure 2). The TBARS measurements shows decrease by about 80% and 40% for oil from lines W92.40 and W92.72, respectively, when compared to control. Also peroxide value is slightly lower for transgenic plant.

Since the flavonoid biosynthesis pathway is separated from fatty acids metabolism, perhaps the way by which the last is activated upon CHS, CHI and DFR overexpression comes from nonspecific antioxidative function of flavonoids. In other words accumulated antioxidative compounds in transgenic seeds partially extract with oil and thus might protect fatty acids against oxidation. The question appeared whether the antioxidants accumulate in oil from transgenic seeds. For that reasons we measured the compound content that might protect fatty acids in oil against oxidation ( Table 2). There was slight but not significant difference in antioxidants level in oil from transgenic seeds compared to control. Even so the stability of oil from transgenic plant is improved. Thus the final conclusion is that fatty acids metabolism changes in transgenic seeds resulted from their protection during synthesis and seed maturation. This conclusion is an agreement with the in vitro studies, where unsaturated fatty acids were protected against oxidation when supplied with micromolar concentration of exogenously added quercetin or β-carotene [32].

**Table 2:** Antioxidants content in flax oil from Linola (control) and transgenic seeds (W92.40 and W92.72) expressed in mg/100 gFW

	Phenolic compounds	Tocopherols	Plastochromanol-8	β-carotene
Linola	1.19 ± 0.04	83.37 ± 0.61	11.48 ± 0.07	0.14 ± 0.01

W92.40	1.27* ± 0.01	83.56 ± 0.72	13.18* ± 0.40	0.16* ± 0.01

W92.72	1.35* ± 0.01	81.15* ± 0.13	11.85 ± 0.21	0.15* ± 0.01

The mean value (n = 6) ± SD for oil from plant cultivated in a field is presented.*Statistically significant (P < 0.05)

#### Biochemical analysis of seedcake extract

**Figure 3:**
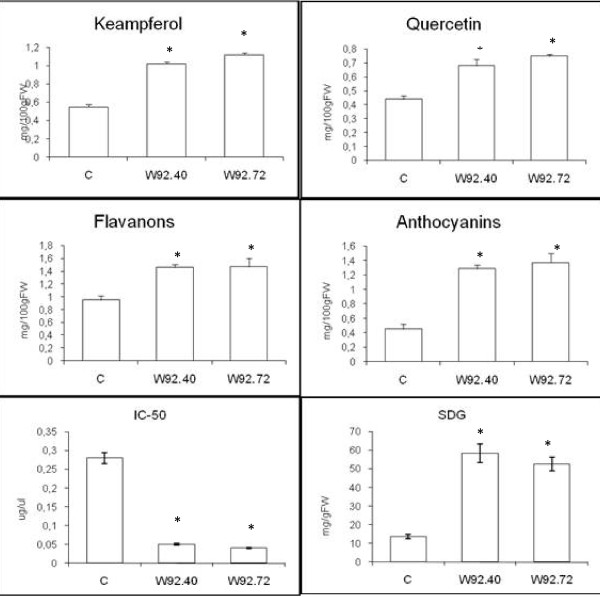
**Biochemical analysis of seedcakes**. The content of kaempferol and quercetin glycoside, total anthocyanins, flavones, SDG content in seedcakes. The antioxidant activity of plant extracts indicated as IC-50 parameter. The control (C) and the transgenic flax plants (numbered). The mean value (n = 6) ± SD for seedcakes from plant cultivated in a field is presented.*Statistically significant (P < 0.05)

Since the changes in fatty acids composition and yield of oil were detected it was reasonable to analyze the residual seed tissues (seedcake) after oil pressing. It was expected an accumulation of antioxidants from flavonoid pathway in seedcakes and thus protection of fatty acids. The analysis was conducted on similar methanol extract as it was performed for seeds. The data presented in Figure 3 shows the significant increase of flavanones and flavonols as well as anthocyanins level. Similar to seed extract, the flavanone content in seedcake extract from W92.40 and W92.72 plant was increased by 53% and 55%, respectively. Also the flavonols level significantly increased in seedcake extract from transgenic lines and was 72% and 85% higher for W92.40 and W92.72, respectively, when compared to control.

We observe about three fold increase of the anthocyanin level in both transgenic lines compared to control.

**Figure 4:**
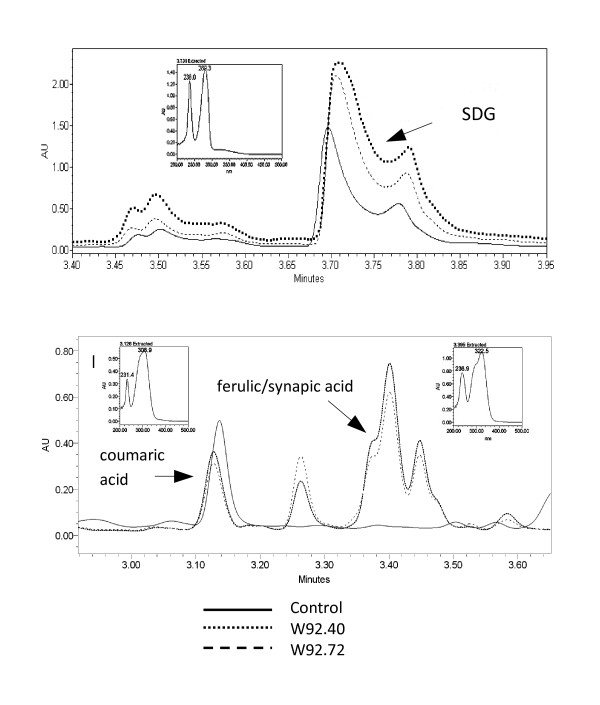
**UPLC chromatograms of a crude extracts of flaxseed**. A. 280 nm chromatogram - SDG identification, B-320 nm chromatogram- phenolic acids identification; spotted line - transgenic line W92.40, dotted line - transgenic line W92.72,solid line - flax control plants.

As a result of flavonoids increase the antioxidant capacity increased significantly (IC50 decrease). The IC50 parameter for seedcake extract from transgenic lines was over 6-fold lower than for control plant. Since this parameter might be also affected by the compounds not extracted with methanol we have decided to analyze antioxidative metabolites in seedcake after alkali hydrolysis. To our surprise UPLC analysis of alkali hydrolyzed seedcake from transgenic plant reveals significant increase of phenolic acids and lignans when compared to control (Figure 4). Of several peaks in UPLC chromatogram we were able to identify coumaric acid and ferulic/synapic acids based on retention time and UV spectra of respective standards. The last might contains ferulic acid and also synapic acid since at chromatographic condition used, retention time of both is quite close (3.376 for ferulic and 3.396 for synapic acids) and their spectra are very similar.

Since phenolic acids (e.g. coumaric) are the precursor of lignan synthesis we have also measured this compound in alkali hydrolyzed extract. It was found that the seedcake extract from transgenic plant show 4.3 fold and 3.8 fold higher secoisolariciresinol diglucoside (SDG) content in transgenic lines W92.40 and W92.72, respectively. Secoisolariciresinol aglycone (4.5 retention time) was also identified only a trace amount compared to SDG quantity was detected (not included in chromatogram).

Phenolic acids and lignans likelihood flavonoids are the main constituents of phenylpropanoid pathway. In view of the presented results there should be operating mechanism of their biosynthesis coregulation based perhaps on substrate availability or prooxidative function of accumulated antioxidants which is discussed in Discussion section.

#### Flax retting and fiber isolation

**Figure 5:**
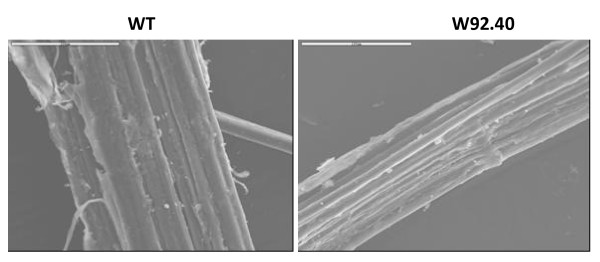
**The SEM analysis of fibres of control (C) and transgenic plants (W92.40)**.

Retting is the process by which linen fibres are obtained. In this process, bast fibre bundles are separated from the core, the epidermis and the cuticle. This is accomplished by the cleavage of pectins and hemicellulose in the flax cell wall, mainly due to the action of plant pathogens. For a high quality of fibre, the proper degree of retting is crucial [33,34] Thus, the efficiency of retting was carried out by scanning electron microscopy (SEM). The SEM of fibres depicted in Figure 5 indicates that retting process is not fully completed in both control and transgenic fibres. The elementary fibres stick together and non-fibrous tissue and cuticle fragments are still present however in case of transgenic fibres the retting is more advanced. The reasons for this could be either the reduction in the pectin content or degree of lignifications or a different arrangement of lignocelluloses polymers in transgenic fibres. In order to verify these suggestions biochemical and spectral analysis of fibres was performed.

#### Biochemical analysis of fibers

Cellulose is the basic structural component of flax fibres. The other important component for fibre structure is lignin, which provides rigidity to the plant. Incrustation of the cell wall by lignin hardens it and reduces its water content, which results in a lower elasticity. Pectins, the other fibre constituents, are mainly responsible for binding single fibres into bundles, and are predominantly released during retting. However, they are also present in the primary wall of individual fibres, and therefore are constituents of the fibres themselves [33]. We determined the content of these compounds in transgenic flax fibres and compared them to the contents for the control fibres. There were no changes in cellulose and pectin compounds content and slight decrease in lignin content compared to fibres from control plants (Table 3). Although the lignin content was lower in transgenic fibres there was no visible changes in scanning electron microscopy of transgenic fibres compared to control (Figure 5).

**Table 3:** Fibre components content in flax fibre Linola (control) and transgenic (W92/40 and W92/72) in ug/mgDW

	Cellulose	Pectin	Lignin
Linola	578.87 ± 48.8	0.025 ± 0.002	2.13 ± 0.04

W92/40	552.42 ± 39.5	0.024 ± 0.001	1.72* ± 0.02

W92/72	622.46* ± 5.51	0.025 ± 0.001	1.76* ± 0.016

The mean value (n = 6) ± SD for oil from plant cultivated in a field in 2008 is presented.*Statistically significant (P < 0.05)

**Figure 6:**
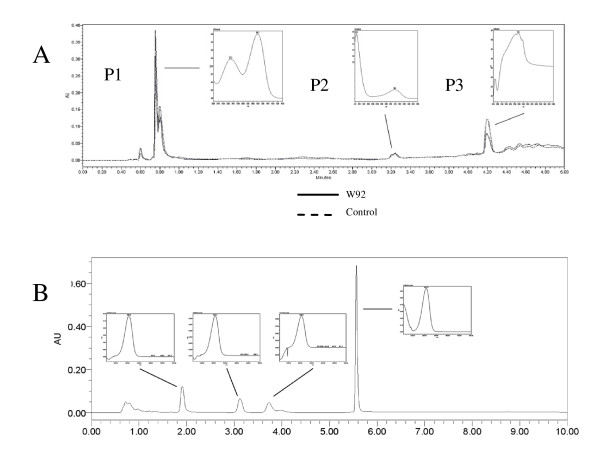
**Separation of methanol extracted components from fibres from control and transgenic plants by UPLC**. A. Separation of components on C18 column, the chromatogram was registered at 280 nm. B. The separation of the main component ( first peak) by HILIC chromatography. The chromatogram was registered at 308 nm.

Since the dramatic changes in flavonoid compounds content in seeds, oil and seedcakes from transgenic plant were detected it was reasonable to analyze the fibers which is also valuable product of flax plant. For the construction of transgenic plants the strong and nonspecific 35S CaMV promoter was used, which resulted in changes occurring in whole plant body. It was expected an accumulation of antioxidants from phenylpropanoid pathway also in fibres. The analysis was as first conducted on methanol extract similarly to this performed for seeds. The data presented in Figure 6 indicate the presence of compounds which can be fairly well identified as derivatives of syringaldehyde and catechine based on retention time and UV spectra and the level of last is lower in transgenic fibres when compared to control. The highest peak in the UPLC chromatogram (P1) showed the UV spectra very similar to coumaric acid/syringic acid standards but their retention time is significantly moved toward lower RT. Thus HILIC chromatography has been applied to separate the constituents of this peak. The HILIC chromatography indicates four peaks and the highest showed UV spectrum identical with syringic acid standard and other three typical for coumaric acid standard. In all cases the retention time however does not fit to the respective standards. Therefore it is suggested that coumaric and syringic derivatives as well as catechine are the main constituents of flax fibres methanol extract and their content in transgenic fibres are lower than in case of fibres from control plant. The HILIC chromatography does not separate catechine and syringaldehyde which localizes at 0.8 min RT.

**Figure 7:**
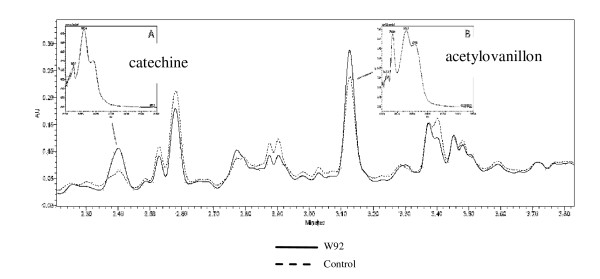
**UPLC Identification of compounds in NaOH hydrolisate of flax fibres obtained from W92 and control plants**. Chromatogram was registered at 280 nm.

For the complete analysis of fibres their alkali hydrolysis was performed which might allow us to identify and analyze the compounds bound to cell well. The result of UPLC analysis of alkali treated fibres is presented in Figure 7. The analysis basically revealed the presence of the same compounds that have been found in fibre methanol extract. However the level of catechine is almost two fold higher in fibre from transgenic plant compared to control. Acetylovanillone is the compound that has been found only in fibre alkali hydrolyzed and its level appeared to be higher in transgenic fibres when comparing to control. The level of phenolic acid (ferulic) in hydrolyzed fibre from transgenic plant is lower than from control.

Since the biochemical analysis of oil, seedcake extract from transgenic seed and fibres reveals the presence of valuable compounds it was of interest from biotechnological point of view to perform detailed structural analysis of those product. In order to do these FT-IR studies of flax products was conducted.

#### Infrared study of flax products

Biochemical studies of oil, seedcake extract and fibre from transgenic flax revealed the changes in level and composition of fatty acids in oil and presence of several compounds of phenylpropanoid origin (kaempferol, coumaric and ferulic/synapic acids, lignans) that were overproduced upon plant transformation with three key genes of this pathway. The significant increase in the quantity of phenolic acids and lignans was detected. In order to verify this data and expand compounds identification IR analysis of flax products were conducted.

The presented assignments of bands to the respective normal modes have been performed by comparison of our own results with published vibrational spectra of the respective standards molecule [35-39]. All spectra were characterized by bands wavenumber and band intensity

It should be noted that the spectra of all analyzed products (oils and seedcake extracts and fibres) from control and transgenic plant were recorded at the same conditions, i.e. using the same dishes, transmission windows and distance washers.

### IR spectra of oil

**Figure 8:**
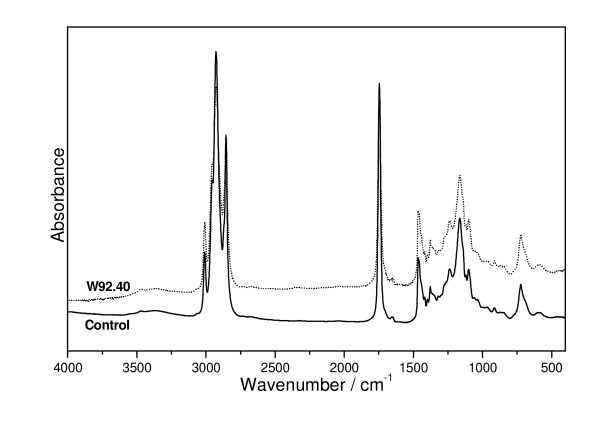
**The IR spectra of the flaxseeds oils obtained from the natural Linola (control) flax and transgenic W92.40**.

The IR spectra of oil from control and transgenic plant has been recorded (Figure 8) and compared to the standard molecules spectra. Their detailed analysis has been performed in our previous paper [40]. The spectra of oil have been integrated to the maxima of the bands at 2928 and 1747 cm^-1^. The former corresponds to the ν_as_(CH_2_) and the later to the ν(C = O) stretching vibrations. They are characteristic for the glycerol ester of the fatty acids and the intensities of these bands can be used as reference points for the IR studies. This approach allows for quantitative comparison of bands intensities in the spectra of oil isolated from control and transgenic plant.

The IR spectra of oil from control and transgenic plant are similar both in respect to the wavenumbers and intensities of the strongest bands. However, the contours observed for the oil from transgenic plant are broader and contain several additional components.

**Figure 9:**
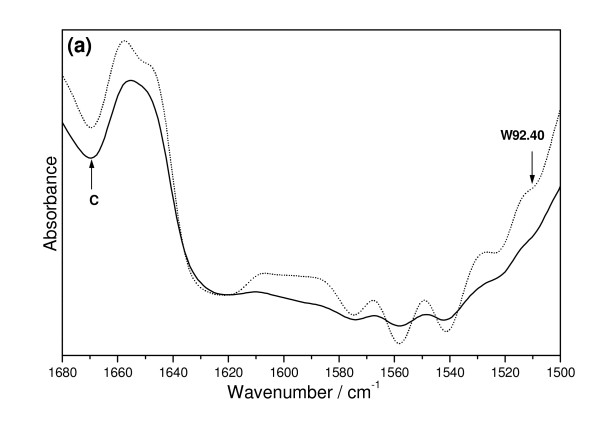
**The IR contours from the region 1500 - 1680 cm-1 of oils from control(C) and transgenic (W92.40) flax plants**. The 1657 cm-1 component is assigned to the ν(C = C) vibration in synapic acid, ferulic acids and kaempferol. The band at 1608 cm-1 fits to standard spectra for secoisolariciresinol (SECO), secoisolariciresinol diglucoside (SDG) and coumaric acid. The 1589 cm-1 band is characteristic for ferulic, coumaric and synapic acids. Bands at 1567 cm-1 correspond to kaempferol, at 1512 -1515 cm-1 to the SECO, SDG and coumaric acid and 1517 cm-1 to synapic acid and SECO.

The differences between the IR spectral contours of oil from control and transgenic flax become evident when very weak bands in some regions are multiplied by factor ten (Figure 9a). All bands of IR contour in the region 1500 - 1680 cm^-1 ^exhibit higher intensity for the transgenic sample. Furthermore the band detected at about 1650 cm^-1 ^for the control oil splits into two components at 1648 and 1657 cm^-1 ^for transgenic sample. These bands can be assigned to the ν(C = C) vibrations. In particular, the later component can be assigned to the ν(C = C) vibration in synapic acid, ferulic acids and kaempferol because their respective bands for standards appear at 1663, 1664 and 1662 cm^-1^, respectively. Similar coincidence is observed for the other bands of this region in the spectra of oils. The band at 1608 cm^-1 ^fits well to those observed at 1601, 1604 and 1602 cm^-1 ^for secoisolariciresinol (SECO), secoisolariciresinol diglucoside (SDG) and coumaric acid, respectively and those at 1589 cm^-1 ^correspond to bands at 1591, 1589 and 1583 cm^-1 ^characteristic for ferulic, coumaric and synapic acids, respectively. Bands at 1567 cm^-1 ^correspond to 1569 cm^-1 ^of kaempferol and those at 1512 cm^-1 ^to the 1515 cm^-1 ^of SECO, 1516 cm^-1 ^of SDG, 1512 cm^-1 ^of coumaric acid, 1517 cm^-1 ^of synapic acid and 1517 cm^-1 ^of SECO. These bands are stronger for transgenic sample suggesting the higher content of these compounds in transgenic plant. Biochemical analysis of total phenolic compounds in oil from transgenic plant also suggests their slightly increased level compared to control. Thus the data from IR analysis confirms these from biochemical estimation. Additionally IR spectra analysis helps to identified phenolic constituents of oil which is not possible in biochemical analysis. Therefore it is suggested that IR analysis of oil provides important information on oil characteristic.

The overall conclusion from the IR study of oil is that several compounds of hydrophilic nature passed to the oil upon seed treatment with high pressure under low temperature. Among those compounds are flavonoids, phenolic acids and lignans. Although we are not able to quantify those compounds it is quite clear that their level is higher in oil from transgenic seed when compared to control. The detection of compounds of phenylpropanoid pathway in oil appears to be a good molecular background for protection of fatty acids from transgenic oil against oxidation which was the case as measured in TBARS method.

### IR spectra of seedcake extract

**Figure 10:**
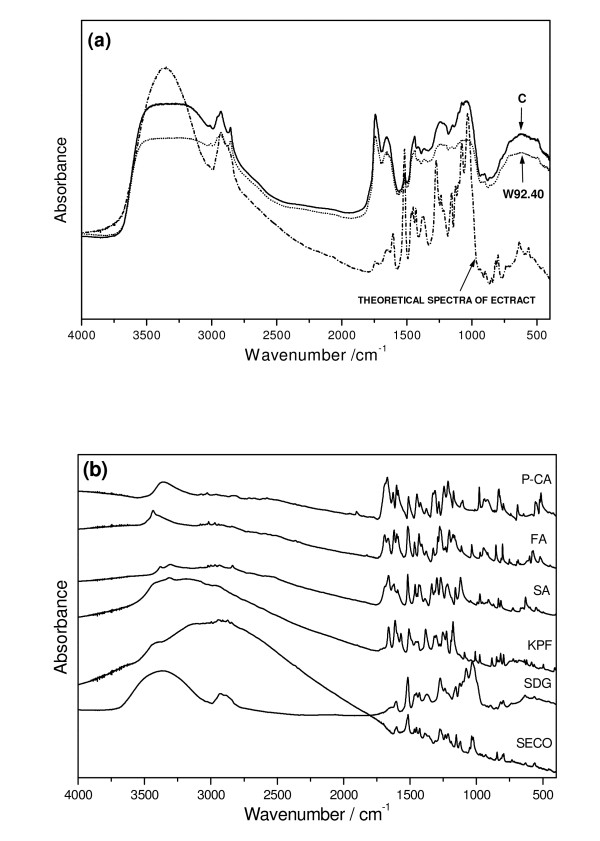
**The IR analysis of seedcake extracts**. The IR spectra of the (a) seedcake extracts obtained from the control(C) and transgenic (W92.40) flax plants; (b) The IR spectra of standards: seicoisolarisiresinole (SECO), seicoisolarisiresinol diglucoside (SDG), kaempferol (KPF), sinapic acid (SA), ferulic acid (FA), p-coumaric acid (P-CA)

**Figure 11:**
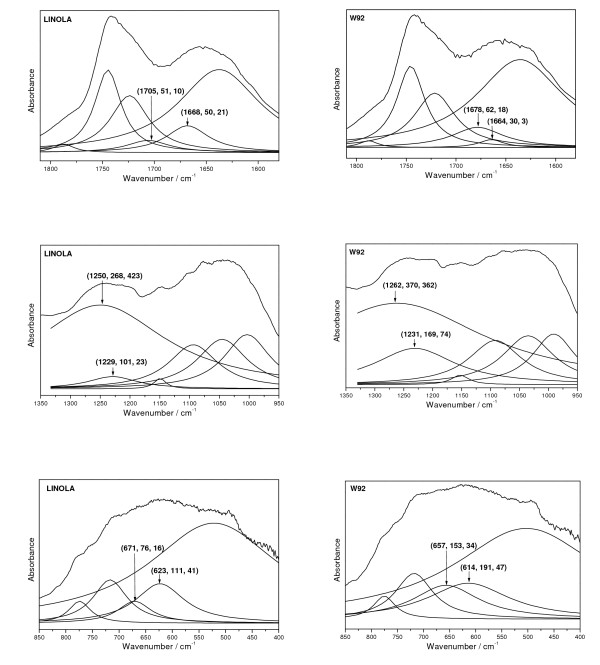
**The deconvolution of the IR contours of control and transgenic (W92) seedcakes extracts**. The deconvolution of the IR contours of control and transgenic (W92) seedcakes extracts in the regions: 1580 - 1800, 950 - 1350 and 400 - 850 cm^-1 ^into Lorenz components. The energetic positions, of the components with differ for the GM and natural flax are given in the parentheses. The 1678 cm^-1 ^band assigned to coumaric acid, 1261 cm^-1 ^band assigned to SECO, 1273 cm^-1 ^to SDG and 1277 cm^-1 ^and 657 cm^-1 ^bands assigned to ferulic acid, and 614 cm^-1 ^that correspond to the bands observed in the region of SDG, kaempferol, coumaric and ferulic acids.

The UPLC analysis of seedcake extract has shown the presence of several compounds with antioxidant activity originated from phenylpropanoid pathway. Among these compounds kaempferol, coumaric, ferulic/synapic acids and lignans were detected. The higher quantity of these compounds in seedcake extract from transgenic plant compared to control was also detected. In order to verify these data IR spectra of seedcake extracts was recorded and compared to respective standard molecules. The spectrum of seedcake extract revealed several bands and they can be grouped into five clear contours that appear in the regions 2000 - 3750, 1580 - 1800, 1310 - 1350, 950 - 1530 and 400 - 850 cm^-1 ^(Figure 10). The contours from the second, fourth and fifth regions emerges vibrations of characteristic groups of phenolic acids, flavonoids and lignans and thus have been deconvoluted into Lorenzian components (Figure 11). Comparing intensity and bandwidth of the bands derived from the deconvoluted contours it is seen that several components have significantly higher intensity and bandwidth for the seedcake extract from transgenic seeds. Beside, some of these bands change their energetic position. Such changes appear for the IR components with the maximum at 1678, 1262, 1231, 657 and 614 cm^-1^. These wavenumbers agree well with the strong intensity bands observed in the IR spectra for the standard molecules. It concerns the following bands:

• 1678 cm^-1 ^that coincides with the strong bands at 1686 and 1671 cm^-1 ^of the coumaric acid,

• 1261 cm^-1 ^that coincides with the strong bands at 1266 and 1275 cm^-1 ^of SECO, 1273 cm^-1 ^of SDG and 1277 cm^-1 ^of the ferulic acid,

• 657 cm^-1 ^that agrees with the bands at 667 cm^-1 ^of the ferulic acid,

• 614 cm^-1 ^that corresponds to the bands observed in the region of SDG, coumaric and ferulic acids and kaempferol.

The observed changes in the energetic position of bands characteristic for phenylpropanoids compounds in IR spectrum of seedcake extract from transgenic seed compared to control confirm the higher content of these compounds in extract from transgenic seedcake which was detected by UPLC analysis. Thus the conclusion is that the extract from transgenic seedcake contains the same phenylpropanoid compounds that were identified by UPLC analysis and they are in higher quantity when compared to control. Further the IR spectra were helpful in identification of phenolic acids (ferulic vs. synapic) which was impossible in UPLC analysis. Thus the IR spectra are suitable for detailed compounds analysis of seedcake extract.

### IR spectra of fibres

FT-IR spectroscopy has been found to be very suitable to detect the major chemical components of flax stems *in vivo *[41]. The technique was also found to provide information on the molecular changes in flax fibres caused by ageing [42], mechanical processing and chemical treatment [36,43]. Therefore, the molecular characteristic of the fibres was further verified by IR spectroscopy.

**Figure 12:**
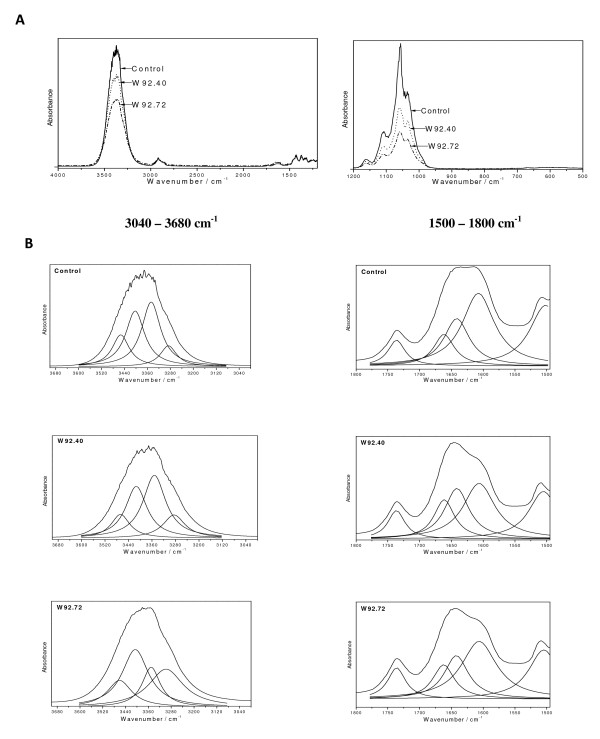
**The IR analysis of fibres**. (A) The IR spectra of the flax fibres obtained from the control and transgenic flax (W92.40, W92.72) (B) The deconvolution of the IR contours of control and transgenic (W92) flax fibres in the regions: 3040 - 3680 and 1500 - 1800 cm^-1 ^into Lorenz components.The broad absorption band at 3400 cm^-1 ^corresponds to the stretching ν(OH) mode of the free hydroxyl groups and those involved in the intra- and inter-molecular hydrogen bonds. The 1400 - 1800 cm^-1 ^region of IR spectra characterize the pectin and lignin constituents of fibres. The component at about 1736 cm^-1 ^corresponds to the ν_as_(COO) vibrations of unconjugated carboxyl group of pectins. The position of band at 1662 cm^-1 ^and 1608 and 1504 cm^-1 ^are characteristic for lignins.

Figure 12a shows the IR Fourier spectra of fibres isolated from the control and transgenic plants. The broad absorption band at 3400 cm^-1 ^corresponds to the stretching νOH) mode of the free hydroxyl groups and those involved in the intra- and inter-molecular hydrogen bonds. The shape of this band is nearly the same for all the samples studied (i.e. the control and transgenic fibres) and very similar to that for purified cotton cellulose, but the bands differ in terms of their absorption intensity. The band could be deconvoluted into four Lorenzian components (Figure 12b) at 3460 (I_(1)_), 3410 (I_(2)_), 3350 (I_(3)_)and 3300(I_(4)_) cm^-1^. The number of components concurs with that expected for the four hydroxyl types of the β-D-glucopyranosyl polymer, which contains three hydroxyls in the glucopyranose ring and one hydroxyl group at the ends of the chains. The integral intensity of these components for the control flax fibres changes in the following direction I_(3) _> I_(2) _> I_(1) _> I_(4) _and for transgenic fibres W92.40 and W92.72: I_(3) _> I_(2) _> I_(4) _> I_(1) _and I_(2) _> I_(4) _> I_(3) _> I_(1)_, respectively. The overall intensity of all the components for the investigated samples follows the direction I_Control _> I_W92.72 _> I_W92.40_. The changes in the intensity of the 3400 cm^-1 ^band components for the control and transgenic fibres probably resulted from different conformations of the intramolecular and intermolecular hydrogen bonds O - H O of the glucopyranose system.

Such changes are expected when different rotary isomers appears in the skeleton of the cellulose polymer, since they differ in the strength and orientation of the hydrogen bond. This leads to the disordered arrangement of the pyranoid rings in the cellulose polymers in fibres from transgenic flax. It should be pointed out that opposite effect has been observed for fibres from transgenic flax producing polyhydroxybutyrate. In this case the intensity of 3400 cm^-1 ^band components was significantly higher than those for control fibres [44,45]

It has commonly been accepted that the most diagnostic regions that characterize the pectin and lignin constituents of fibres is 1400 - 1800 cm^-1 ^region of IR spectra. The bands in this region of IR spectra for control and transgenic W92.40 and W92.72 flax fibres could be deconvoluted into five Lorenzian components that approximate well the envelope of these contours.

**Figure 13:**
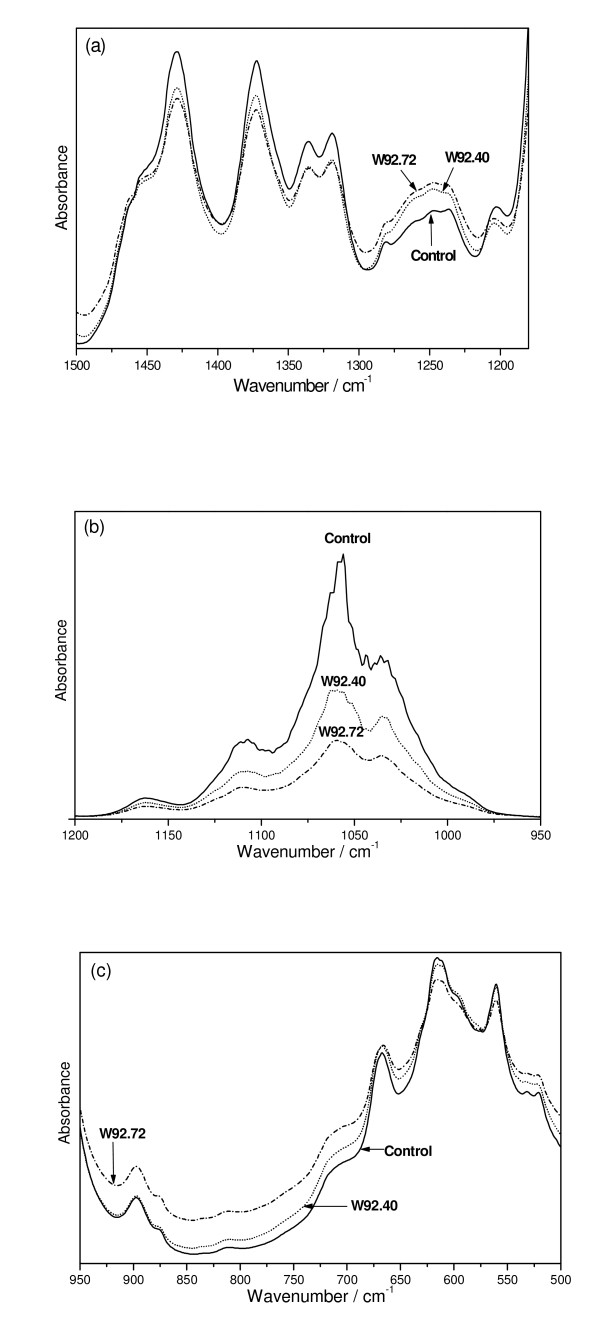
**The deconvolution of the IR contours of fibres**. The IR contours in the region (a) 1500 - 1200 cm^-1^, (b) 1200 - 950 cm^-1 ^and (c) 950 - 500 cm^-1 ^of the control and transgenic (W92.72, W92.40) flax fibres. All this regions are typical for flax cellulose consisting of some amounts of lignins and pectins.

The component at about 1736 cm^-1 ^corresponds to the ν_as_(COO) vibrations of unconjugated carboxyl group of pectins. The relative intensity of this band fulfills the relations: I_c _< I_72 _< I_40 _showing the irregular dependence on the pectin content (I_40 _was expected to be smaller than I_72_), although the GM samples exhibit somewhat their greater content. The position of band at 1662 cm^-1 ^is characteristic for lignins and its intensity increases for W92.40 and W92.72. This band has been considered as originating from both protein impurity and water associated with lignin [46]. The irregular trend is observed for the band at 1642 cm^-1 ^that corresponds to the ν_as_(COO) vibration of the conjugated carboxyl group. Its intensity clearly increases for the W92.40 but decreases for W92.72 samples. On the other hand, the bands at 1608 and 1504 cm^-1 ^show regular behavior i.e. I_c _> I_40 _> I_70 _but their integral intensities are practically very close. They correspond to the stretching vibrations of the aromatic skeleton of the lignins [46].The contours observed in the IR spectra of the flax fibres in the regions 1200 - 1500, 950 - 1200 cm^-1 ^and 500 - 950 cm^-1 ^are typical for the flax cellulose consisting of some amounts of lignins and pectins (Figure 13). The bands from these multiplets can be assigned to the vibration of δ_as_(CH_3_,CH_2_) at 1429 cm^-1^, δ_s_(CH_3_,CH_2_) at 1372 cm^-1^, δ(CH) at 1319 and 1336 cm^-1^, ν(C-C) and ν(C-O) at the range from 1200 to 1300 cm^-1^, δ(φ-OH) at 1163 cm^-1^, ν_as_(C-O-C) at the range of 1000 - 1110 cm^-1^, γ(CH) at the range of 850 - 1000 cm^-1^, and δ(θ) at the range from 500 to 720 cm^-1^.

The highest difference between control and transgenic fibres appeared at the two regions from 1200 to 1300 cm^-1 ^and from 1025 to 1125 cm^-1^. The first corresponds to the in-plane stretching vibrations of pyranoid rings coupled with the in-plane bending of the OH·····O bonds and the last to the ν_as_(C-O-C) vibrations.

The δ(OH·····O) bands at about 1250 cm^-1 ^exhibit reverse intensity to the ν(OH···O) vibration observed at 3400 cm^-1 ^which suggests that the amount of the hydrogen bonds is different in the control and transgenic flax fibres and, therefore, a coupling between the elementary fibres from transgenic plant is weaker than in control. The substantial decrease in the intensity of the contour at about 1050 cm^-1 ^for the transgenic fibre suggests that the cellulose polymers are shorter than for control because they contain less C-O-C bridges.

The data from IR study leads to the conclusion that cellulose polymer of transgenic fibres are shorter and more loosely bound than those from control plant.

## Discussion

Flax (*Linum usitatissimum *L.) is an important source of natural fibres in several temperate regions of the world. Flax oil has long been used in human and animal diet and in industry as a source of oil and as the basal component or additive of various paints or polymers. Recently there has been a growing interest in the probiotic properties of flax and in its beneficial effects on coronary heart disease, some kinds of cancer and neurological and hormonal disorders [1-3]. The beneficial effects are mostly due to flax lipids. Flax oil is the richest plant source of linoleic and linolenic polyunsaturated fatty acids (PUFA), which are essential for humans since they cannot be synthesized in the organism and must be ingested with food. However, essential fatty acids are highly susceptible to oxidation and therefore flax oil has a very short shelf life.

The seedcake, the residual tissues after oil pressing, has scarcely been used only for animal feeding and some beneficial effect on animal growth was observed [47]. There is no report on seedcake biochemical composition and the effect of tissue components on accumulation and composition of fatty acids. Also there is no report on potential industrial application of biochemical components from seedcake.

Flax fibres derive from the phloem, and can be separated from the inner bark outside the cambium by the process called retting [34] For many years, flax fibres were mainly used for textile production. Now however, they are used as constituents of composite material for high-tech applications [48]. The strong, lightweight and low-cost flax fibres are used instead of conventional glass-fibres in reinforced plastic manufacture. Since they are biodegradable, their final composite product is easily recyclable. Very recently flax fibres application in biomedicine is also under investigation. Their beneficial role as wound dressing has been reported [7]

In order to improve oil stability and to expand seedcake and fibre application, mainly in medicine, transgenic flax plant has been generated and analyzed. It was expected that overexpression of three genes coding for key enzymes of flavonoid route will result in accumulation of broad spectrum of antioxidant molecules in each part of plant and thus making their basic products (oil, fibres) biotechnologically more valuable.

The initial step in the flavonoid route of the phenylpropanoid pathway is the synthesis of naringenin chalcone through condensation of three malonyl-CoA units with *p*-coumaroyl-CoA. The reaction is catalyzed by the action of chalcone synthase (CHS). The naringenin chalcone is a branch point for all flavonoid biosynthesis [49]. Chalcones are very labile compounds and could be accumulated as dihydrochalcones or rapidly converted to the colorless flavanone naringenin by the chalcone isomerase (CHI). Flavanones act as a precursor for the synthesis of flavones and 3-OH-flavanones (dihydroflavonols). Dihydroflavonols can then be converted into flavonols by FLS (flavonol synthase) or into anthocyanins by DFR (dihydroflavonol 4-reductase).

Since our gene construction that has been introduced into transgenic flax contained cDNAs for CHS, CHI and DFR enzyme, the flavonols and anthocyanins compound content was measured. The data obtained showed an increase in these compounds level in flaxseeds, seedcakes and fibres from plants of transgenic lines. It should be pointed out that measured flavonoid compounds in seed could be than found in seedcake extract and thus suggesting that the residual tissues after oil extraction from seeds appeared to be suitable source of biomedical valuable compounds. It is also important to notice that generated transgenic flax are stable in respect to expression of exogenously introduced genes since the field grown plants of third generation contained all transgenes and they are active which was confirmed by PCR analysis of transgenic plant with total mRNA as template.

Also interesting is the identification of increased quantity of phenolic acids and lignans in seedcake from transgenic seed. It is known that these compounds like flavonoids are the constituents of phenylpropanoid pathway however they are biosynthesized on different metabolic route. In view of the presented results there should be mechanism in operation coordinating their biosynthetic coregulation [50].

The core phenylpropanoid pathway converts phenylalanine to p-coumaric acid. Several branches radiate from this core reaction. Early branch point leads to the formation of simple phenylpropanoids like phenolic acids. The later incorporation of three malonyl-CoA molecules leads to generation of flavonoids. Lignans are dimers and lignins are the polymer of monolignol alcohol. Monolignol forms from coumaric acid, oxygen and methyl groups incorporated into the aromatic ring. This intermediate is then thought to be converted to respective aldehyde and then to the monolignol (coumaryl, ferulyl and sinapyl alcohols). The way in which lignans (dimer) and lignin (polymer) are formed from monolignol precursor is still poorly understood. Early suggestion was that the final stage of monolignol polymerization resulted from random coupling of monolignol units that did not require the involvement of enzymes. This opinion is now modified, it is evidenced that monolignol in the form of glycosides are transported to cell wall and then oxidized to form radicals that combine to produce polymer. There are several classes of enzymes present in cell wall that could catalyze monolignol oxidation including peroxidases, laccases, polyphenol oxidases and dirigent protein. Lignin polymer can be formed artificially by adding either peroxidase together with H_2_O_2 _or laccase with O_2 _which serve as oxidizing agent. This is the most substantial evidence that the same process might operating *in vivo *and suggests that monolignol dimers and polymers can be formed by random chemical interactions as well as by enzymatic control.

It is as yet unknown how these compounds (lignans) biosynthesis is regulated upon flavonoids overproduction [25]. We speculate that strong activation of flavonoids synthesis in transgenic flax and compounds accumulation might be the reason for this. The high concentrations of antioxidants may result in pro-oxidative activity. Thus the increase in antioxidative compound concentration upon CHS/CHI/DFR overexpression might result in local promoting of coumaric acid oxidation and monolignol radicals generation. This suggestion is supported by the finding that the level of coumaric acid in seedcake extract from control plant is about 30% lower than this from transgenic plant.

Thus, the seedcake from transgenic seed appears as a richest source of SDG. It is important to notice the beneficial role of this compound for human health. There are several reports concluding the role of SDG in protection against different types of cancer [49,51,52]. SDG can be metabolized by the colonic microflora to the mammalian lignans, enterodiol and enterolactone. That is the reason why flax lignans exhibit weak estrogenic and antiestrogenic properties, in a tissue-specific manner, and have potential role in the prevention and treatment of breast cancer and other hormone dependent cancers [53,54]. A great number of animal model and human studies suggest that a higher intake of lignans reduces the risk of many chronic diseases including cardiovascular diseases and all types of diabetes [55-58]

Both flavonoids and phenolic acids were shown to exhibit antioxidant properties [11]. Since there was an increase in those compounds content detected in transgenic plants, an increase in antioxidant capacity was expected. Indeed the extract from transgenic seed and seedcake showed almost the same IC50 value and this was far lower than for control. Thus the accumulation of compounds from phenylpropanoid pathway mainly occurs in seedcake and strongly affects antioxidant potential of transgenic product.

It is known that flavonoids due to their hydrophilic nature only very partially coextract with the oil during its production. Even so the accumulation of antioxidants in seedcake was expected to affect fatty acid composition. Indeed oil produced from transgenic seeds contains more unsaturated fatty acids and the total level of fatty acids was also increased. Thus the higher quantity of phenylpropanoid compounds in seedcake indirectly affect the fatty acids stability perhaps by protection them against oxidation during technological process of oil production.

Flax retting is the process of fibre isolation and was faster in case of transgenic plant. In retting, bast fibre bundles are separated from the core, the epidermis and the cuticle [33]. This is accomplished by the cleavage of pectins and hemicellulose in the flax cell wall, a process mainly carried out by plant pathogens like filamentous fungi [34]. The retting efficiency depends on the degree of lignification. Since reduced lignin content in transgenic flax was observed the easier retting was expected. This was however not the case. Scanning electron microscopy does not show differences between transgenic and control fibres. Biochemical analysis of fibres suggests the increased level of catechine and acetylovanillone. Although the first resulted from flavonoid biosynthesis enhancement the second presumably derives from lignin degradation [56] The decrease in lignin content in fibres from transgenic plant agreed with this. Also decrease in the content of syringaldehyde in transgenic fibres which is the product of lignocellulose degradation [56] confirm that degradation process might occurs as more advanced in transgenic fibres than in control.

Infrared spectroscopy has been used for molecular characteristic of flax products for it was found to be very suitable for identification of the major chemical components and also found to provide information on the molecular changes in flax fibres caused by ageing, mechanical processing and chemical treatment [34,41].Thus the main products of flax plant (oil, seedcake extract and fibres) were further analysed by this method and data compared to those from biochemical analysis.

The IR study of oil revealed that several compounds of hydrophilic nature coextract with the oil upon seed treatment with high pressure under low temperature. Among those compounds are flavonoids, phenolic acids and lignans. Although the method does not provide quantitative data on compounds content it is quite clear that their level is higher in oil from transgenic seed when compared to control. The detection of compounds of phenylpropanoid pathway in oil appears to be a good molecular background for protection of fatty acids from transgenic oil against oxidation which agrees with data from oil analyzed in TBARS method.

The observed changes in the intensity of bands characteristic for phenylpropanoids compounds in IR spectrum of seedcake extract from transgenic seed compared to control confirm the higher content of these compounds in extract from transgenic seedcake which was detected by UPLC analysis. Thus the conclusion is that the extract from transgenic seedcake contains the same phenylpropanoid compounds that were identified by UPLC analysis and they are in higher quantity when compared to control. Further the IR spectra helped to identify phenolic acids (ferulic vs. synapic) which were not resolved on UPLC column. Thus the IR spectra are suitable for detailed compounds analysis of seedcake extract.

The data from IR study confirmed the results of the biochemical analyses of the flax fibres and additionally suggests that arrangement of the cellulose polymer in the transgenic fibres differed from that in the control and a significant decrease in the number of hydrogen bonds was detected.

Thus the overall conclusion from the IR study of flax main product is that several compounds of phenylpropanoid pathway are accumulated in oil, seedcake and fibres upon three genes overexpression resulting in production of stable oil and fibres and seedcake extract ready for use in biomedicine.

## Conclusions

The overall conclusion of this work is that the main product of flax (seeds, oil and fibres) from transgenic plant overexpressing three key genes from flavonoids route resulted in accumulation of several flavonoids, phenolic acids and lignans. All these compounds have an antioxidative nature and thus suggesting the use of modified fibres in production of fabrics for medicine (e.g. wound dressing), stable oil enriched in fatty acids for human diet and seedcakes for extraction of compounds with potential biomedical application. Their application in biomedicine is under investigation. Very recently those fabrics were successfully used in chronic wound therapy [7] To our best knowledge this is the first report describing the potential of all products from genetically engineered flax cultivated in semi-technical scale for wide biomedical application.

## Competing interests

The authors declare that they have no competing interests.

## Authors' contributions

MZ-carried out the biochemical analysis of seeds and seedcakes, drafted the manuscript. AK - carried out fibre biochemical analysis and participated in writing of the manuscript, LD- carried out IR analysis of seedcakes and oil, KS- carried out IR analysis of fibres, AP-participated in oil analysis, JH-participates in interpretation of IR analysis and conceived of IR part of study, JS- conceived of the study and participate in its design and coordination. All authors read and approved the final manuscript.
