# Correction: Flavonoid engineering of flax potentiate its biotechnological application

**DOI:** 10.1186/1472-6750-12-47

**Published:** 2012-08-07

**Authors:** Magdalena Żuk, Anna Kulma, Lucyna Dymińska, Katarzyna Szołtysek, Anna Prescha, Jerzy Hanuza, Jan Szopa

**Affiliations:** 1 Faculty of Biotechnology, University of Wrocław, Wrocław, Poland; 2 Institute of Chemistry and Food Technology, Faculty of Economics and Engineering, University of Economics, Wrocław, Poland; 3 Department of Food Science and Nutrition, Wroclaw Medical University, Nankiera 1, 50-140, Wrocław, Poland; 4 Institute of Low Temperatures and Structure Research, Polish Academy of Sciences, Wrocław, Poland; 5 Linum Fundation, Stablowicka 147/149, 54-066, Wroclaw, Poland

## 

Following publication of our paper in BMC Biotechnology [1] it came to light that numerous sections of text were similar or identical to our previous publications. We acknowledge that portions of the text in the Background, Methods, Results and Discussion of the article published in BMC Biotechnology (above) are identical to wording in our previous publications in Spectrochimica Acta (SAA) [2], Biotechnology Progress [3] and Physiological and Molecular Plant Pathology (PMPP)[4] and that these publications should have been correctly cited.
